# Human immunodeficiency virus-1 core: The Trojan horse in virus–host interaction

**DOI:** 10.3389/fmicb.2022.1002476

**Published:** 2022-08-29

**Authors:** Wei Wang, Yan Li, Zhe Zhang, Wei Wei

**Affiliations:** ^1^Institute of Virology and AIDS Research, The First Hospital of Jilin University, Changchun, Jilin, China; ^2^Key Laboratory of Organ Regeneration and Transplantation of Ministry of Education, Institute of Translational Medicine, First Hospital, Jilin University, Changchun, Jilin, China

**Keywords:** HIV, core, capsid, host factors, innate immunity

## Abstract

Human immunodeficiency virus-1 (HIV-1) is the major cause of acquired immunodeficiency syndrome (AIDs) worldwide. In HIV-1 infection, innate immunity is the first defensive line for immune recognition and viral clearance to ensure the normal biological function of the host cell and body health. Under the strong selected pressure generated by the human body over thousands of years, HIV has evolved strategies to counteract and deceive the innate immune system into completing its lifecycle. Recently, several studies have demonstrated that HIV capsid core which is thought to be a protector of the cone structure of genomic RNA, also plays an essential role in escaping innate immunity surveillance. This mini-review summarizes the function of capsid in viral immune evasion, and the comprehensive elucidation of capsid-host cell innate immunity interaction could promote our understanding of HIV-1’s pathogenic mechanism and provide insights for HIV-1 treatment in clinical therapy.

## Introduction

Human immunodeficiency virus (HIV) is the major pathogen accounting for human immunodeficiency syndrome, and innate immunity is the first defensive line against pathogenic infection (Mogensen, 2009; Yin et al., 2020). The genic single-strand RNA (ssRNA), double-strands DNA (dsDNA) transcripted from ssRNA or viral components of HIV can act as pathogen-associated molecular patterns (PAMPs) that are recognized by pattern recognition receptors (PRRs) on the host cell. PAMPs stimulate an immediate immune response and activate the antiviral cascade to clear intra- and extra-cellular virions (Le Sage et al., 2014). During HIV-1 infection, PAMPs of each stage could be recognized by different specific receptors. In the entry and uncoating stage, genomic ssRNA could be detected by the RIG-I-like receptors (RLRs) family [retinoic acid-inducible gene I (RIG-1) and melanoma-differentiation-associated protein 5 (MDA5)] and Toll-like receptors (TLR) including TLR7 and TLR8 (Gringhuis et al., 2010; Muenchhoff et al., 2014; Altfeld and Gale, 2015; Meas et al., 2020). While the innate immune activation by HIV RNA is controversial, whether other uncharacterized RNA sensors are responsible for sensing HIV-1 intron-containing RNA remains to be determined (Akiyama et al., 2018; McCauley et al., 2018). Recent studies demonstrated that HIV-1 recruits cellular 2′-O-methyltransferase FTSJ3, leading to 2′-O-methylation of viral RNA and thereby escaping MDA5-dependent innate immune sensing in host cells (Ringeard et al., 2019). After entry, HIV-1 transcriptional product could be recognized by interferon (IFN)-inducible protein 16 (IFI16), DEAD-box helicase (DDX) family, and cyclic GMP-AMP synthase (cGAS). Additionally, cGAS recognizes HIV-1 in the integration- and translation-stages (Brai et al., 2020; Siddiqui and Yamashita, 2021). After immune activation, the host cell induces antiviral signaling pathways to drive robust IFN, proinflammatory cytokines, and chemokines production for efficiently inhibiting HIV-1’s replication (Thompson et al., 2014; Hoang and Paiardini, 2019). In addition to modulation of antiviral signaling pathways, the restriction factors such as Serine Incorporator family (SERINC; Rosa et al., 2015; Usami et al., 2015), apolipoprotein B mRNA editing enzyme catalytic subunit 3 (APOBEC3s; Sheehy et al., 2002), tripartite motif-containing protein 5α (Trim5α; Sayah et al., 2004; Stremlau et al., 2004; Pertel et al., 2011), dGTP-dependent deoxynucleotide triphosphohydrolase SAM domain, HD domain-containing protein 1 (SAMHD1) and BST-2/tetherin limit viral reproduction and spread through their unique manners (Neil et al., 2008; Hultquist and Harris, 2009; Goldstone et al., 2011; Hrecka et al., 2011; Laguette et al., 2011; Su et al., 2014; Yin et al., 2020).

Under strong selection pressure, HIV has evolved strategies to evade immune surveillance for its successful replication. The HIV-1 accessory proteins involved in immune evasion have been thoroughly researched for many years. For example, Nef can exclude SERINC 3 and SERINC 5 from HIV virions (Jin et al., 2020), while, Vif induces the degradation of APOBEC3s by usurping the host CRL5 E3 ligase (Yu et al., 2003). Meanwhile, Vpr targets host cell restriction factors, such as helicase-like transcription factor (HLTF), tet methylcytosine dioxygenase 2 (TET2) or nuclear factor-kappa B (NF-κB) signaling pathway to facilitate viral replication (Wang and Su, 2019; Yan et al., 2019; Khan et al., 2020). Furthermore, Vpx, the homologous protein of simian immunodeficiency virus (SIV) has the same ability to selectively inhibit cGAS activity by targeting functional domain of stimulator of IFN genes (STING) and triggers SAMHD1 degradation (Hrecka et al., 2011; Laguette et al., 2011; Hofmann et al., 2012; Wei et al., 2012; Su et al., 2019). Additionally, HIV Vpu could counteract the antiviral effects from BST-2 and also exhibits potent to inhibit IFN response by acting on NF-κB signaling pathway (Van Damme et al., 2008; Douglas et al., 2009; Iwabu et al., 2009; Langer et al., 2019). Thus, in HIV infection, the unstructured accessory proteins are broadly utilized to resist host innate immunity through multifaceted strategies. However, the anti-innate immunity of HIV-1’s structure protein—the capsid (CA) protein has been barely reported. In recent studies, CA has been identified as a restriction factor that participates in anti-innate immune response during HIV-1’s lifecycle. Here, we review the function of CA in HIV immune evasion, throughout understanding of interaction between HIV CA protein and host cell innate immune surveillance will make HIV’s pathogenic mechanism clearly, and also provide new sight for fighting AIDS disease in clinical therapy.

## Human immunodeficiency virus capsid

During virus maturation, HIV structural polyprotein Gag synthesized in the cytoplasm is proteolyzed by protease (PR) into four domains-matrix (MA), CA, nucleocapsid (NC) and P6 with two short peptides-SP1 and SP2 (Ono et al., 2004), ~ 250 CA formed hexamers and 12 pentamers assembling into the fullerene-like, cone-shaped core structure to enclose viral ribonucleoprotein complex and associated proteins internally (Yang et al., 2012). CA composed of 231 residues folds into two domains at both ends of the peptide chain through a flexible linker (residues 146–150; Pornillos et al., 2009; Rossi et al., 2021), N-terminal domain of CA (NTDs) which contains seven α helices and a cyclophilin A (CypA)-binding loop forms hexamers through an NTD-NTD interface (Gamble et al., 1996), C-terminal domain of CA (CTDs) forms dimers with adjacent hexamers through a CTD–CTD interface (Gamble et al., 1997). The NTD–CTD interface formed by NTD and CTD from two subunits is a crucial mechanical element for generating lattice curvature, the inner structure formed by adjacent CA proteins provides a platform for binding host cell factors to evade immune sensors and complete the viral lifecycle (Bhattacharya et al., 2014). Hexamers formed by CA interaction are linked together by N-terminal residues of CA-CTD, and the lattice structure is stabilized through intra-CA monomers modulated by water molecules (Gres et al., 2015). Some amino acid residues in the CA of pentamers have been confirmed to interact with hexamers, and pentamers are localized at high curvature domains, suggesting that hexamers may form the high curvature for pentamers to insert or the pentamers themselves supply a high angle of curvature to maintain the stability of mature core. Moreover, maintaining the stability of the CA core is essential for viral biological functions, alteration of stability would dramatically reduce HIV-1 infectivity (Pornillos et al., 2011; Mattei et al., 2016; Dharan et al., 2017; Achuthan et al., 2019). In the past decade, CA core was thought to be merely a container for viral RNA, but recently, multiple roles of CA during infection have been identified. Importantly, CA-related immune evasion has attracted considerable attention in many research fields, and CA inhibitors have been thought to be a potential antiviral target. Therefore, the exact role of CA in protecting HIV-1 from the innate immune activity is discussed below.

### Capsid protects HIV from cGAS sensor

In HIV infection, cGAS is the best-studied receptor in host innate immune responses (Gao et al., 2013; Herzner et al., 2015; Sumner et al., 2020). At first, cGAS protein is recruited to the CA in a polyglutamine-binding protein-1 (PQBP1) or Non-POU (Pit-Oct-Unc) domain-containing octamer-binding protein (NONO)-dependent manner in the cytosol and nucleus respectively, enabling cGAS molecules to recognize the reverse transcriptional product of HIV (Yoh et al., 2015, 2022; Lahaye et al., 2018). Subsequently, cGAS binds to viral dsDNA and produces a second messenger 2′3′cGAMP. 2′3′cGAMP detected by ER-resident STING and triggers STING translocation to Golgi, activating TANK-binding kinase 1 (TBK1), leading to phosphorylation of IFN regulatory factor 3 (IRF3) which is subsequently translocated into the nucleus to activate the expression of IFN and IFN-stimulated genes to resist HIV infection (Siddiqui et al., 2019; Hopfner and Hornung, 2020). Additionally, the TLR and canonical Hippo signaling pathway enhance cGAS sensing of HIV to efficiently inhibit viral replication (Siddiqui and Yamashita, 2021).

**Figure 1:**
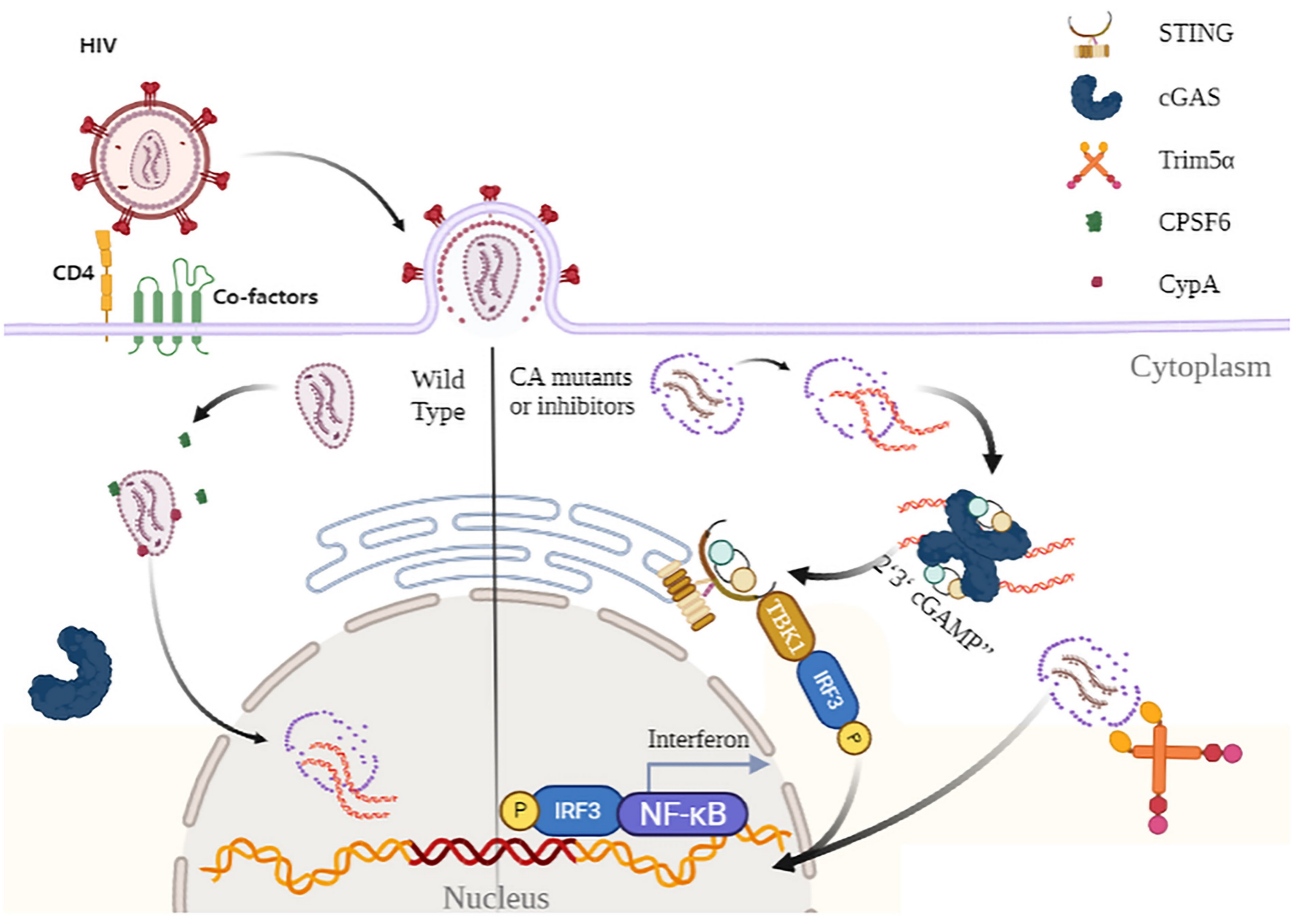
The schematic of HIV-1’s innate immune evasion in CA-dependent pathway. After the invasion, CA protects the HIV-1 virus after the invasion from innate immune recognition and ensures it translocates into the nucleus to complete its lifecycle. Alteration of CA formation and CA- interaction by genetic mutation or CA inhibitor could induce host cell IFN secretion through the cGAS-STING signal pathway. Disrupting CA interaction with CypA, CPSF6 will stimulate NF-κB activity, and downstream IFN response leads to defects in HIV-1’s infectivity. Furthermore, CA-CypA interaction is essential for counteracting with Trim5α to keep the CA core intact and inhibit Trim5α-induced immune response through the NF-κB signal pathway.

During HIV infection, the viral CA core must maintain its stability to ensure an efficient infection. CA-derived core structure is required to protect the reverse transcription complex (RTC) and pre-integration complex (PIC) from degradation by host restriction factors (Francis and Melikyan, 2018). However, recent study has demonstrated a new function of HIV-1 CA, suggesting that CA protein plays an essential role in evading cGAS monitoring (Sumner et al., 2020). Disrupting CA formation through inhibition of HIV Gag cleavage by genetic manipulation (Gag mutant L363I M367I) or lopinavir (a protease inhibitor) activates the cGAS signaling pathway and significantly reduces HIV infectivity in PMA-treated THP-1 and U87 cells. Transcriptional upregulation of IFN-stimulated genes downstream of cGAS (C–X–C motif chemokine ligand 10, IFN-induced protein with tetratricopeptide repeats 2, and Myxoma resistance protein A) has been observed in PMA-treated THP-1 cells, thus leading to a strong IFN response after lopinavir treatment (Sumner et al., 2020). Similarly, small molecule inhibitor PF-74 causes IFN-stimulated gene activation in wildtype and MAVS^−/−^ cells, but not in cGAS^−/−^ cells, suggesting that malformation of CA structure induces an innate immune response *via* a cGAS-dependent pathway (Kumar et al., 2018; Siddiqui et al., 2019; Sumner et al., 2020). Importantly, HIV-1 infection does not trigger a cGAS-mediated immune response (Cingoz and Goff, 2019; Elsner et al., 2020). While HIV-2 fails to evade the immune recognition in macrophages and dendritic cells, mainly because of distinct features of the CAs of HIV-1 and HIV-2 (Lahaye et al., 2013). The innate immune sensor of the HIV CA, NONO, binds to the HIV-2 CA with more affinity than HIV-1 in the nucleus, enabling sensing of HIV DNA and subsequent activativation of cGAS-STING pathway (Lahaye et al., 2018). HIV-1’s CA-dependent immune evasion strategy, where CA cloaks viral DNA from cGAS sensing successfully explains this phenomenon (Figure 1).

### Capsid-mediated viral dependent factors

During HIV-1’s lifecycle, multiple virus-dependent factors of the host cell could be hijacked at different steps to promote viral replication through CA-dependent methods, some of which are manipulated to evade the immune response. Virus-dependent factors that participate in immune evasion are introduced below.

### Cleavage and polyadenylation specificity factor subunit 6

Cleavage and polyadenylation specificity factor subunit 6 (CPSF6), a component of the cleavage factor 1 (CFIm), interacts with CA protein and is strictly conserved in lentiviruses (Lee et al., 2010; Saito et al., 2020). CPSF6 binds to HIV-1 CA through the CTD of CA (Iwabu et al., 2009), and assists HIV-1 infection during the uncoating steps (Saito and Yamashita, 2021). The interaction between CPSF6 and HIV-1 CA is essential for successful viral replication. Depleting CPSF6 induces strong IFN activity, suggesting CPSF6 provides an advantage to HIV-1 for avoiding immune activation. Compared to HIV-1 wildtype virus, depleting connection between HIV-1 and CPSF6 using HIV-1 CA mutants (N74D) is lethal for viral replication in primary human monocyte-derived macrophages (MDM). N74D mutant triggers IRF3 and RelA nuclear translocation, which activates the NF-κB signaling pathway and ultimately stimulates strong IFN production to suppress viral replication (Sumner et al., 2020). Moreover, depleting the CPSF6-CA interaction leads to HIV-1 RTC and PIC accumulating at the nuclear envelope in macrophages, while the IFN secretion induced by retention of many subviral complexes in the cytoplasm also results in poor infectivity (Bejarano et al., 2019).

### Cyclophilin A

Cyclophilin A (CypA) belongs to the cellular cyclophilin family, essential for viral replication in a variety of viruses, such as hepatitis C virus (HCV), coronaviruses (CoVs) and HIV, and its polymorphisms affect viral susceptibility and disease progression (Le Sage et al., 2014; Mamatis et al., 2022). CypA interacts with CypA binding loops at the N-terminal of HIV-1 CA, as described above. The CypA-CA binding is crucial for HIV-1 replication in primary human macrophages and dendritic cells, HIV-1 mutant deficiency in CypA-CA binding stimulates type I IFN secretion and induces an antiviral state. In MDM infection, the HIV-1 mutant P90A impairs CA-CypA interaction, which stimulates IFN-β secretion and activates the immune response, resulting in efficient suppression of viral infection (Sumner et al., 2020). In a recent study, a CypA inhibitors, cyclosporine (CsA), and a non-immunosuppressive analog of CsA, SmBz-CsA could alter the CypA-CA affinity and elicit extreme antiviral activity in MDM, they also induce robust IFN-β production and completely inhibit HIV-1 infection (Li et al., 2009; Sumner et al., 2020). From the above, it can be observed HIV-1 CA-CypA interaction is essential for viral replication through protecting itself from innate immune recognition, however, the excellent anti-HIV-1 activity of CypA inhibitors *in vitro* is a promising prospect for clinical medicine development (Carnes et al., 2018). Moreover, CypA could counteract Trim5α during HIV-1 infection, the exact function of CypA facilitating HIV-1 to evade the Trim5α restriction is discussed later (Li et al., 2016).

### Nuclear pore components

The nuclear pore component or nucleoporin (NUP), a component of the nuclear pore complex, could be manipulated by HIV-1 during its nuclear import stage. Among the NUP family, HIV-1 CA directly interacts with NUP153 and NUP 358, whereas the other NUPs are thought to serve as co-factors by genome-wide RNA interference screens (Kane et al., 2018). Depletion of CA interaction with NUP 153 and NUP 358 by HIV-1 mutants N74D or P90A leads to a strong IFN production, reducing viral replication in MDM because NUP-CA interaction could efficiently block nuclear translocation of IRF3 and NF-κB (Gres et al., 2015). However, some studies have proposed that NUP-CA interaction is not the determining factor for immunosuppression, and the immune activation caused by HIV-1 mutants is ascribed to a defect of recruitment of CPSF6 through NUP358. Whether NUPs directly modulate immune responses remains controversial, thus; the exact mechanism of NUPs in immune evasion requires further study (Rasaiyaah et al., 2013; Shen et al., 2021).

Myxovirus resistance protein B (MXB/MX2), a dynamin-like GTPase, inhibits infection of HIV and primate lentivirus by blocking their trafficking and nuclear entry steps (Goujon et al., 2013; Kane et al., 2013; Liu et al., 2013; Wei et al., 2016; Yamashita and Engelman, 2017; Betancor et al., 2019, 2021). After the invasion, NUP153 and NUP 358 are manipulated by HIV-1 to resist the IFN-induced MXB reaction to complete its lifecycle (Liu et al., 2013; Fribourgh et al., 2014). Except for NUP153 and NUP158, other NUP factors such as NUP62, NUP88, NUP93, NUP153, NUP214, and NUP358 have been identified as HIV-1 co-factors in recent studies, which block MXB recognition and assist HIV-1 in completing its nuclear transportation through different NUP-dependent pathway (Engelman, 2021). It means that, if necessary, multiple NUP factors could be employed by HIV-1 to evade MXB surveillance (Kane et al., 2018). Accordingly, it is easy to elucidate why HIV-1 epidemic clades in different regions exhibit variant characteristics of MXB-resistant activity (Kane et al., 2018; Rossi et al., 2021).

### Trim5α

Trim5α is a non-human primate restriction factor that disrupts reverse transcription and blocks viral infection during early infection (Sayah et al., 2004; Berthoux et al., 2005; Novikova et al., 2019). HIV-1 CA is captured by the SPRY domain of polymerized Trim5α and causes premature uncoating, where the replicated elements inside the cone-shaped core are exposed to host cell’s cytoplasm and the reverse transcription terminated. Although Trim5α exhibits a strong antiviral activity immediately after viral infection, it is a pity that, the interaction between CA and its co-factors results in the inability of human Trim5α (huTrim5α) to bind to HIV-1 CA directly; thus, its suppression of HIV-1 replication is weak (Kim et al., 2019). Compared to that of the wild-type virus, the HIV-1 mutant N74D bearing deficiencies in CPSF6 and CypA-interaction exhibits infectivity defect, whereas knockout of huTrim5α rescues infectivity of N74D in human CD4^+^ T cells, but depletion of CPSF6 does not affect HIV-1 reverse transcription, meaning that CPSF6 is not a key modulator against huTrim5α restriction. Otherwise, the small molecule inhibitor CsA and GS-Cyp Ai3 targeting CypA-CA interaction decrease HIV-1 infectivity in macrophages and CD4^+^ T cells, respectively (Kim et al., 2019), suggesting that CypA is an anti-HIV-1 target for huTrim5α (Ganser-Pornillos and Pornillos, 2019; Selyutina et al., 2022b). Moreover, in HIV-1 P90A mutant infection, Trim5α acts as PRR to induce IFN-β production through NF-κB and AP1 signaling pathway in a non-canonical autophagy-dependent method (Saha et al., 2020), which means intact CA-CypA is pivotal for resisting multiple innate antiviral pathways induced by huTrim5α in HIV-1 infection. Although the CypA-CA interaction protects HIV-1 from the restriction of Trim5α, CsA which has been previously used in clinical trials targeting CypA-CA, exhibits efficient anti-HIV-1 activity by triggering pre-mature uncoating and innate immune responses (McArthur et al., 2019; Link et al., 2020). This evidence demonstrates that Trim5α and CypA-CA interaction is a promising anti-HIV target for further anti-HIV-1 medical development.

### Disrupting stress granule

After viral infection, the virus-induced production of reactive oxygen species and HIV-1 protease activity increase the intracellular stress, which leads to the assembly of translationally silent ribonucleoprotein and proteins into stress granule (SG; Le Sage et al., 2014; Rao et al., 2018). Stress granule formation suppresses viral replication, and therefore, HIV-1 manipulates multiple factors to disrupt SG production. The N-terminal CA of Gag disrupts canonical type I SGs through interaction with the host eukaryotic elongation factor 2 (eEF2; Valiente-Echeverria et al., 2014), and non-canonical type II SGs are blocked by Gag through disrupting hypophosphorylated 4EBP1 by targeting eIF4E (Cinti et al., 2016). Moreover, the interaction between CypA with the N-terminal of Gag is essential for the immune sensing in monocyte-derived dendritic cells. Importantly, CypA-CA binding mutant HIV-1 G89A and CA inhibitor CsA cause HIV-1 losing the ability of modulating SG production (Manel et al., 2010; Le Sage et al., 2014; Cinti et al., 2016).

## Discussion

As an extracellular intruder, HIV-1 needs to minimize the cellular processes that induce innate immune responses to complete its lifecycle. From the membrane to the nucleus, the virus must undergo a long-range movement to arrive at its destination and cloak itself perfectly to avoid PRR recognition (Sabo et al., 2013). Under the selected pressure, HIV-1 has evolved unique strategies to counteract the innate immune system to complete its successful infection. The genetic material, accessory proteins, and replication-associated elements (PAMPs) are packed inside the CA core to deceive cGAS recognition. Additionally, the accessory protein, including Vpr, Vpu, Vif, Vpx, and Nef are packaged into the CA-derived core before its budding, to assist HIV in evading immune surveillance, which makes the proper core stability import for HIV-1 infection by keeping these accessory protein safely inside, and the host cell-dependent factors strictly binds to CA to counteract Trim5α for maintaining the stability of the viral structure (Rose et al., 2021). HIV-1 depends on CA-derived core to avoid the immune sensors encountered in host cytoplasm during its early infection, which is consistent with a recent study that CA core maintains its intact structure and escorts HIV-1 into the nucleus for its efficient infection, and increasing evidence demonstrated that an intact or nearly intact CA core passes through nuclear pore complexes to enter the nucleus, suggesting that HIV-1 CA provides unique strategies for protecting HIV-1 from innate immune recognition (Christensen et al., 2020; Shen et al., 2021).

Because of CA’s important role in immune evasion, antiviral research targeting CA protein attracts increasing attentions from scientists and pharmaceutical institutions. Many small-molecule inhibitors have been developed and exhibit efficient antiviral activity *in vitro* and clinical trials. GS-CA and GS-62072, developed from PF74, disrupt the CA interaction with CPSF6 and NUP153, enhancing innate immune responses and ultimately eliminating the HIV-1; additionally, the long-acting activity makes them suitable for clinical treatment (Carnes et al., 2018). However, some epidemic variants breakthrough PF74 inhibition independent of reduction of PF74 binding, two SIVs that are not sensitive to PF74 and GS-CA have been recently identified (Siddiqui et al., 2019; Twizerimana et al., 2020). The newly emerged immune evasion strategies of HIV-1 transmitted variant makes it difficult to develop broad-spectrum anti-HIV-1 medicine, but additional studies targeting diverse HIV-1variants may uncover distinct mechanisms to escape restriction by these CA inhibitors (Twizerimana et al., 2020; Saito and Yamashita, 2021). Altough conformational changes induced by CA-mutant decrease the sensitivity to CA inhibitor (Selyutina et al., 2022a), and anti-HIV activity of CA inhibitor strictly dependent on cell types (Twizerimana et al., 2020), it is promising that further study of CA-related immune evasion will clearly eluciated HIV-1’s pathogenic mechanism and provide a theoretical basis for antiviral therapeutics.

## Author contributions

WWe conceptualized the ideas. WWa performed the literature search and drafted the original manuscript, and drew the figure. WWe, YL, and ZZ revised the manuscript. All authors contributed to the article and approved the submitted version.

## Funding

This work was supported by the National Natural Science Foundation of China (81772183 and 31800150), the National Major Project for Infectious Disease Control and Prevention (2018ZX10731-101-001-016), the Department of Science and Technology of Jilin Province (nos. 20190304033YY and 20180101127JC), the Open Project of Key Laboratory of Organ Regeneration and Transplantation, Ministry of Education, the Program for JLU Science and Technology Innovative Research Team (2017TD-08), and Fundamental Research Funds for the Central Universities.

## Conflict of interest

The authors declare that the research was conducted in the absence of any commercial or financial relationships that could be construed as a potential conflict of interest.

## Publisher’s note

All claims expressed in this article are solely those of the authors and do not necessarily represent those of their affiliated organizations, or those of the publisher, the editors and the reviewers. Any product that may be evaluated in this article, or claim that may be made by its manufacturer, is not guaranteed or endorsed by the publisher.
